# Role of artificial intelligence in pediatric intensive care: a survey of healthcare staff perspectives in Saudi Arabia

**DOI:** 10.3389/fped.2025.1533877

**Published:** 2025-02-24

**Authors:** Khouloud Abdulrhman Al-Sofyani

**Affiliations:** ^1^ Department of Pediatric, Pediatric Critical Care Unit, Faculty of Medicine, King Abdulaziz University, Jeddah, Saudi Arabia; ^2^ Clinical Skills and Simulation Center, King Abdulaziz University, Jeddah, Saudi Arabia

**Keywords:** artificial intelligence, pediatric intensive care unit, survey, healthcare, ethical concerns

## Abstract

**Background:**

Artificial Intelligence (AI) has the potential to revolutionize Pediatric Intensive Care Units (PICUs) by enhancing diagnostic accuracy, improving patient outcomes, and streamlining routine tasks. However, integrating AI into PICU environments poses significant ethical and data privacy challenges, necessitating effective governance and robust regulatory frameworks to ensure safe and ethical implementation. This study aimed to explore valuable insights into healthcare professionals' current perceptions and readiness to adopt AI in pediatric critical care, highlighting the opportunities and challenges ahead.

**Methods:**

A cross-sectional study conducted an online survey among healthcare practitioners at King Abdulaziz University Hospital in Jeddah, Saudi Arabia. The survey included questions about professional roles, experience, and familiarity with AI, their opinions on AI's role, trust in AI-driven decisions, and ethical and privacy concerns. Statistical analyses were performed using IBM SPSS.

**Results:**

Results found varying familiarity with AI among healthcare professionals, with many expressing limited knowledge of AI applications in PICU settings. Despite this, there was growing recognition of AI's current applications. Trust in AI-driven decisions for PICU management was mixed, with most expressing partial trust. Opinions on AI's role in enhancing diagnostic accuracy and improving patient outcomes varied. Ethical considerations, data privacy, and effective governance to address regulatory and ethical challenges were highlighted as critical concerns.

**Conclusion:**

Healthcare practitioners in the PICU preferred using AI for routine patient monitoring but had concerns about its use in diagnoses and advanced healthcare. Concerns were held regarding data privacy, security breaches, and patient confidentiality.

## Introduction

Artificial Intelligence (AI) is an umbrella term pertaining to the areas of learning algorithms, decision-making, processing natural languages, and robotics (1). It describes all the methods by which a computer system imitates the cognitive functions humans are endowed with, such as logical thinking, reasoning, decision-making, and most importantly, memory or learning from past experiences and incidences (2). Therefore, AI has immense potential to advance health applications, including tertiary care of critical patients, and can be adapted to almost any area of medicine (3–5).

Lately, the use of AI has been gaining importance in the healthcare sector as it offers substantial opportunities to improve healthcare outcomes, reduce costs, and significantly impact society (2). With the robustness of AI, it is likely to have a complementary role to human cognitive capability. However, with the increase in data and further improvement of the model, AI can even exceed human cognition regarding precision and decision-making. However, reliability remains an issue, which has met skepticism in the use of AI in the healthcare sector (6). For example, IBM Watson uses AI algorithms to analyze patients' data and guides physicians in exploring and devising a treatment strategy (7). However, it has come under scrutiny for making risky and unreliable cancer therapy recommendations. Therefore, it is at the physician's discretion to make judicial understandings and correct choices while using AI.

The advent of newer technology and improvement of care standards has considerably raised the quality of medical treatment in critical conditions. Despite this progress, traditional critical care has certain limitations owing to completely understating and addressing the complexities of the patient's health status, timely prediction of deterioration, and providing timely treatment (8, 9). Under such circumstances, the use AI can be utilized to recognize patterns in complex data, suggest the next course of treatment and improve the overall outcome (8). However, the availability of abundant, robust, and accurately prepared AI is of utmost importance in making such models successful (10). Various AI tools have been developed to augment the healthcare sector. The United States Food and Drug Administration (FDA) has approved several AI-based tools, thereby paving the foundation for the inclusion of such tools in the healthcare sector (11).

Pediatric patients are usually exposed to a high risk of fatal decompensation and are sensitive to medicine owing to the developing immune system. Therefore, delay in treatment or slight oversight in dose can complicate the health situation further. Therefore, the clinicians engaged in pediatrics are often exposed to high-pressure situations that demand precision, skills, and decision-making prowess. Under such circumstances, the ability to comprehend large amounts of medical information quickly and efficiently is required to diagnose and develop a treatment plan for a given baby. Nevertheless, physicians and nurses can leverage the capability of AI for fast-paced, accurate decision-making in pediatric intensive care units (PICUs). The first recorded evidence of the use of AI in PICU dates to 1968, when SHELP a computer-assisted medical decision-making system was used to diagnose inborn errors of metabolism in children (12). Machine learning and artificial intelligence are crucial in overcoming challenges in defining and subclassifying disease phenotypes in pediatric critical care (13). The application of AI in PICUs has come a long way and has been used to detect and as decision support systems for monitoring glucose levels, predicting metabolic disorders, implementing cognitive stimulation therapies, and diagnosing neonatal sepsis, etc (14–17).

Various studies have been undertaken to develop AI-based systems specifically designed for the needs of Neonatal Intensive Care Units (NICUs) and PICUs. These systems primarily focus on predicting critical outcomes, such as mortality and morbidity risks in seriously ill children (15, 18), assessing the effectiveness of medications (19), and estimating the length of hospital stays (20, 21). For example, AI has been employed in whole-genome sequencing to identify genetic disorders and predict mortality and morbidity risks (18). It has demonstrated potential in forecasting the effectiveness of caffeine treatment for apneic episodes in neonates (19), predicting individual physiological states to estimate the length of stay in PICUs (20), and analyzing cardiorespiratory signals to monitor vital signs in NICU patients (22). Furthermore, AI's application in predicting shock in children under the age of 12 represents a noteworthy advancement in pediatric critical care (23).

Although the use of AI in PICU has gained attention to an extent, this is still met with skepticism owing to several problems such as availability, cost-effectiveness, awareness among the health care providers, and educational aspects (24). While AI holds considerable promise for transforming PICUs by enhancing diagnostic accuracy, improving patient outcomes, and streamlining routine tasks, its adoption is still constrained by several barriers. Current literature has primarily concentrated on the technical and clinical advancements of AI, with insufficient attention given to healthcare professionals’ readiness, perceptions, and trust in employing AI, particularly in sensitive contexts like PICUs. Additionally, ethical and data privacy concerns surrounding AI integration have not been thoroughly examined in pediatric critical care, where patient confidentiality and safety are of utmost importance.

Therefore, this study aimed to conduct a survey among the health care providers and gain insight into their perspective on integrating AI into PICU for patient care and better outcomes of clinical conditions. This study explored healthcare professionals' knowledge and familiarity with AI applications in PICUs, focusing on their trust in AI-driven decisions for routine monitoring, diagnostics, and patient management. It also gathered insights on the potential of AI to improve diagnostic accuracy and patient outcomes while highlighting ethical concerns such as data privacy and security. The research assessed the readiness to integrate AI into standard operations and identified barriers like knowledge gaps and infrastructure challenges. Additionally, it examined the diverse views on necessary regulatory frameworks for the safe deployment of AI in PICUs. By understanding these perspectives, the study highlights both the opportunities and challenges of incorporating AI in pediatric healthcare, aiming to generate actionable insights for effective implementation and future policies.

## Methods

### Study population

This cross-sectional study was designed to investigate healthcare practitioners' prior knowledge and opinions regarding the use of AI in the PICU setting at King Abdulaziz University Hospital, Jeddah, Saudi Arabia. The study population included clinicians, physicians, and healthcare providers involved in PICU care at King Abdulaziz University Hospital. An electronic survey was created using the open-source “Google Forms” platform to collect participant views on integrating AI into PICUs for improved patient outcome management.

### Survey design

The survey was designed following the Checklist for Reporting Results of Internet E-Surveys (CHERRIES) guidelines and consisted of 21 questions, including three partially categorized and 18 close-ended questions. The content covered participant demographics, professional roles, and experiences (Q3–Q5); knowledge and perceptions of AI in healthcare and pediatric intensive care units (PICUs) (Q7–Q9); prior use of AI in PICUs (Q10, Q11); opinions on AI applications, trust levels, and routine task assistance preferences (Q12–Q18); and views on outcomes, privacy concerns, and expectations (Q19–Q23). The survey incorporated validated constructs from prior studies to assess both objective knowledge and subjective expectations regarding AI usage. The questionnaire details are provided in Supplementary File 1 to ensure transparency and replicability for future studies.

### Survey distribution, data collection, and ethical considerations

The questionnaire was disseminated to the participants through online platforms while ensuring that their involvement remained completely anonymous and consensual. At the beginning of the questionnaire, a comprehensive preface outlined the study's objectives, providing participants with a clear understanding of its purpose and significance. To maintain the integrity of the data collected, each participant was limited to a single submission.

Upon receiving the consent to participate in the survey, the questionnaire was distributed to all healthcare professionals via a Google Form link, facilitated by the pediatric office. Participants were provided with a written notice highlighting that participation was voluntary, their responses would remain confidential, and no personal information would be collected. Volunteers were instructed to complete the questionnaire, which aimed to assess various dimensions of the AI PICU environment. Responses were automatically saved and securely stored for subsequent analysis. By completing the survey, participants effectively provided their informed consent to contribute to the research. The ethical approval for the study was obtained from the Research Ethics Committee, Unit of Biomedical Ethics, Faculty of Medicine, King Abdulaziz University (HA-02-J-008).

### Statistical analysis

The statistical analysis for this study was conducted using SPSS version 21 (SPSS Inc, Chicago, IL, USA. Data analysis was conducted employing descriptive statistics to summarize response distributions, central tendencies, and variability. Categorical variables were summarized by presenting their frequencies and calculated percentages, while continuous variables were described using their means and standard deviations (SD). To investigate the various factors associated with the implementation of artificial intelligence (AI) in Pediatric Intensive Care Unit (PICU) settings, we utilized independent *t*-tests and chi-square tests, depending on the nature of the data. A *p-*value of <0.05 was established as the threshold for rejecting the null hypothesis, indicating that any observed differences or associations were statistically significant.

## Results

### Demographic characterization of the study population

The demographic characteristics of the participants are presented in Table 1. Responders were mostly women (*n* = 33, 75%) compared to their male counterparts (*n* = 25, 25%). 50% of the participants were in the age group of 30–39, whereas 29.5% were in the age group of 40–49 (Table 1). No participants were beyond the age of 60.

**Table 1:** Background characteristics of the survey participants of the study.

Age of participants	Number of participants	Percentage (%)
20–29	4	9.1
30–39	22	50.0
40–49	13	29.5
50–59	5	11.4
Gender of participants		
Female	33	75.0
Male	11	25.0

### Professional expertise and characteristics of the study population

Healthcare providers who are closely involved in all aspects of a PICU setting were surveyed. The study population mostly included nurses (*n* = 29, 65.9%), followed by doctors (*n* = 11, 25%), and the rest were technicians (2.3%) and managers (2.3%). Regarding professional experience, the participants had an experience of at least 1–9 years (*n* = 19, 43.2%), and 10–19 years (*n* = 17, 38.6%). A total of 8 participants, representing 18.2%, had over 20 years of experience. Furthermore, the majority of our participants (*n* = 35, or 79.5%) were employed by government sector hospitals, while 7 participants (15.9%) worked in private hospitals, and 2 participants (4.5%) were affiliated with both government and private sector hospitals (Table 2).

**Table 2:** Professional experience of the survey participants of the study.

Profession of participants	Number of participants	Percentage (%)
Clinician	2	4.5
Doctor	11	25.0
Manager	1	2.3
Nurse	29	65.9
Technician	1	2.3
Professional experience		
1–9 years	19	43.2
10–19 years	17	38.6
20 and above years	8	18.2
Professional practice		
Government hospitals	35	79.5
Other	2	4.5
Private hospitals	7	15.9

### Characterization of the participants in terms of their familiarity with using the internet and AI in PICU setup

**Figure 1:**
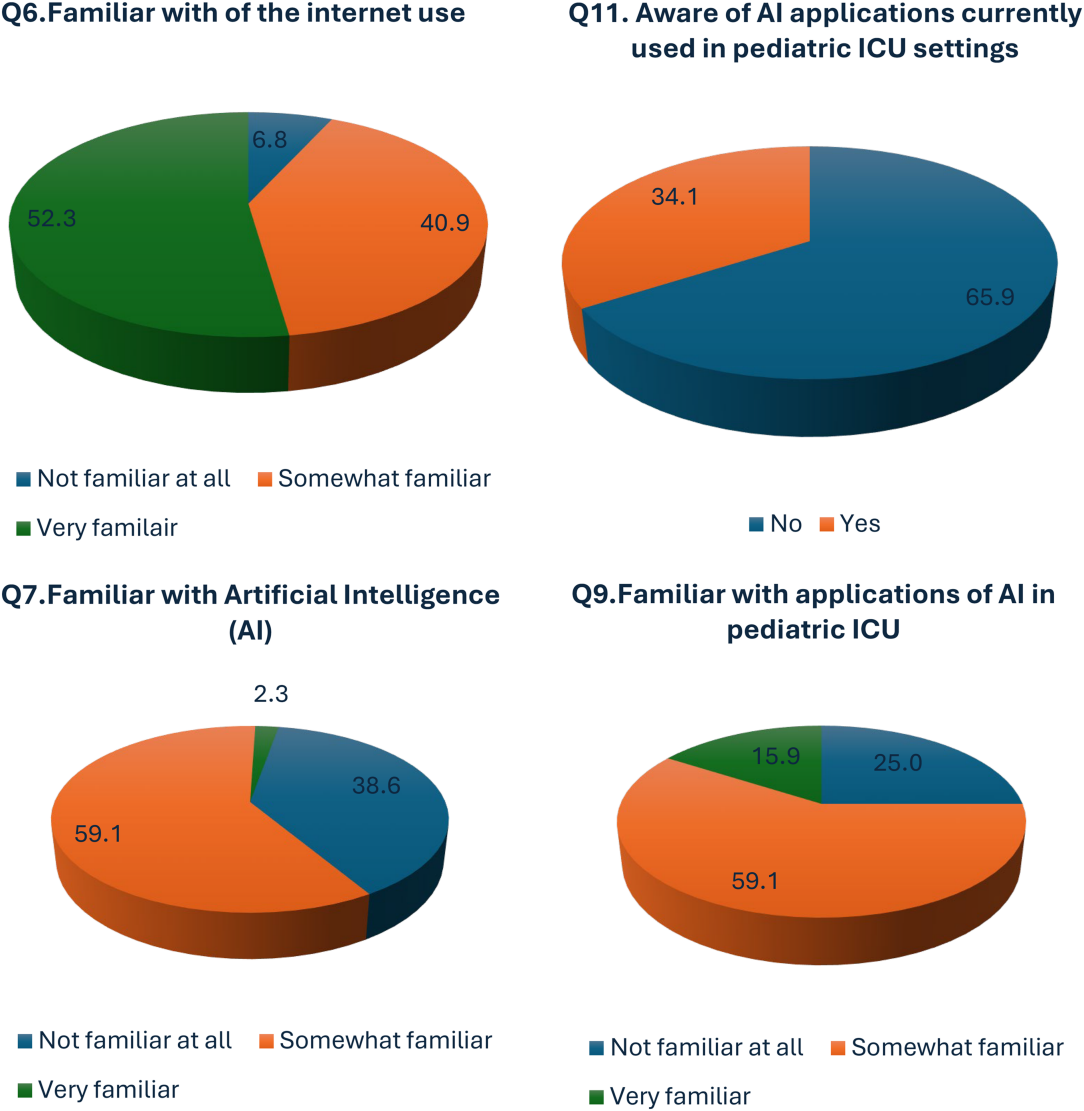
Knowledge of AI adoption in PICU care.

In our study, most of our participants (*n* = 23, 52.3%) were very familiar with the use of the Internet and regularly used it in their day-to-day non-professional activities (Figure 1). On the other hand, 40.9% (*n* = 18) of our participants were somewhat familiar with the Internet and used it in their daily activities. However, 3 of our participants, accounting for 6.8% of the study population, were not familiar with the Internet (Table 3).

**Table 3:** Familiarity with using the internet and AI in PICU setup among healthcare professionals.

Demographic and other variables	Familiar with artificial intelligence		
	No	Yes	Total
Age of respondents			
20–29	0.0%	13.8%	9.1%
30–39	60.0%	44.8%	50.0%
40–49	33.3%	27.6%	29.5%
50–59	6.7%	13.8%	11.4%
Gender			
Female	33.3%	20.7%	25.0%
Male	66.7%	79.3%	75.0%
Profession			
Doctor	33.3%	20.7%	25.0%
Nurse	60.0%	69.0%	65.9%
Clinician	6.7%	3.4%	4.5%
Technician	0.0%	3.4%	2.3%
Manager	0.0%	3.4%	2.3%
Professional experience (in years)			
1–9	46.7%	41.4%	43.2%
10–19	40.0%	37.9%	38.6%
20 and above	13.3%	20.7%	18.2%
Professional practice			
Government hospitals	66.7%	86.2%	79.5%
Other	26.7%	10.3%	15.9%
Private hospitals	6.7%	3.4%	4.5%
Familiar with the use of the internet			
Very familiar	46.7%	55.2%	52.3%
Somewhat familiar	46.7%	37.9%	40.9%
Not familiar at all	6.7%	6.9%	6.8%

With the prior information on familiarity with the use of the Internet, we further assessed the participants' knowledge and willingness to adopt AI in ICU care. Only a small number of participants (2.3%) were familiar with AI, with most participants (59.1%) not familiar at all and a small number (38.6%) somewhat familiar (*p*-value = 0.00) (Figure 1; Supplementary Table 1). A substantial portion of our participants, 59.1%, demonstrated a solid understanding of both AI and its specific applications within PICUs. Notably, even among those who reported limited knowledge of AI as a whole, a significant percentage of participants (15.9%) were nonetheless aware of its practical applications in the PICU context, a finding that yielded a statistically significant (*p*-value = 0.001). Additionally, 34.1% of the participants were aware of the current AI applications in PICU (Figure 1).

### Participant profile based on the willingness to adopt AI in PICU setup

**Figure 2:**
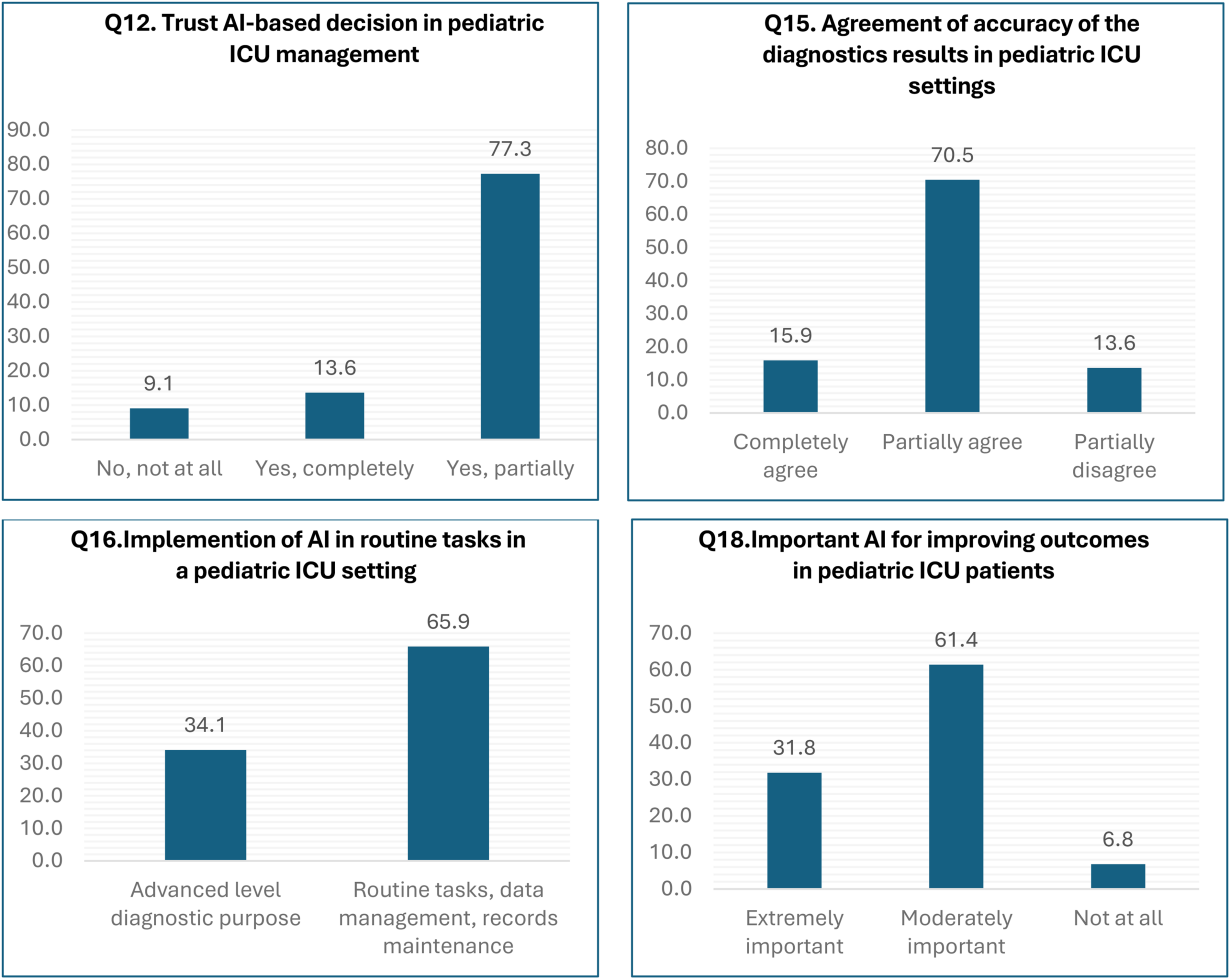
Attitude of AI adoption in PICU care.

A set of questions was intended to investigate the general perceptions regarding implementing AI in the PICU. The majority of our participants (77.3%) were in favor of partially trusting the decision made by AI in pediatric ICU management, while 13.6% placed their complete trust in AI, and the rest 9.1% of the participants were skeptical about the decision of using AI in PICU management (*p-*value = 0.00) (Figure 2; Supplementary Table 1). Similarly, 70.5% of the participants partially agreed with the accuracy of AI-based diagnostic results in the PICU setting, whereas 15.9% completely agreed with the accuracy of AI-based diagnosis, while 13.6% partially disagreed (Figure 2). Furthermore, 65% of the participants favored implementing AI for routine tasks, data management, and records maintenance (*p*-value = 0.05). However, 34.1% of the participants even supported the implementation of AI for advanced-level diagnostic purposes in PICU (Figure 2). Moreover, 6.8% of the participants felt that implementing AI cannot change the outcome of patients in PICU, whereas 61.4% felt that implementing AI can moderately improve the outcome in PICU setup. Nevertheless, a small number of participants i.e., 31.8%, believed that AI can drastically improve the outcomes of patients in PICU (*p*-value = 0.05) (Figure 2).

### Ethical concerns and patient data privacy in AI implementation

**Figure 3:**
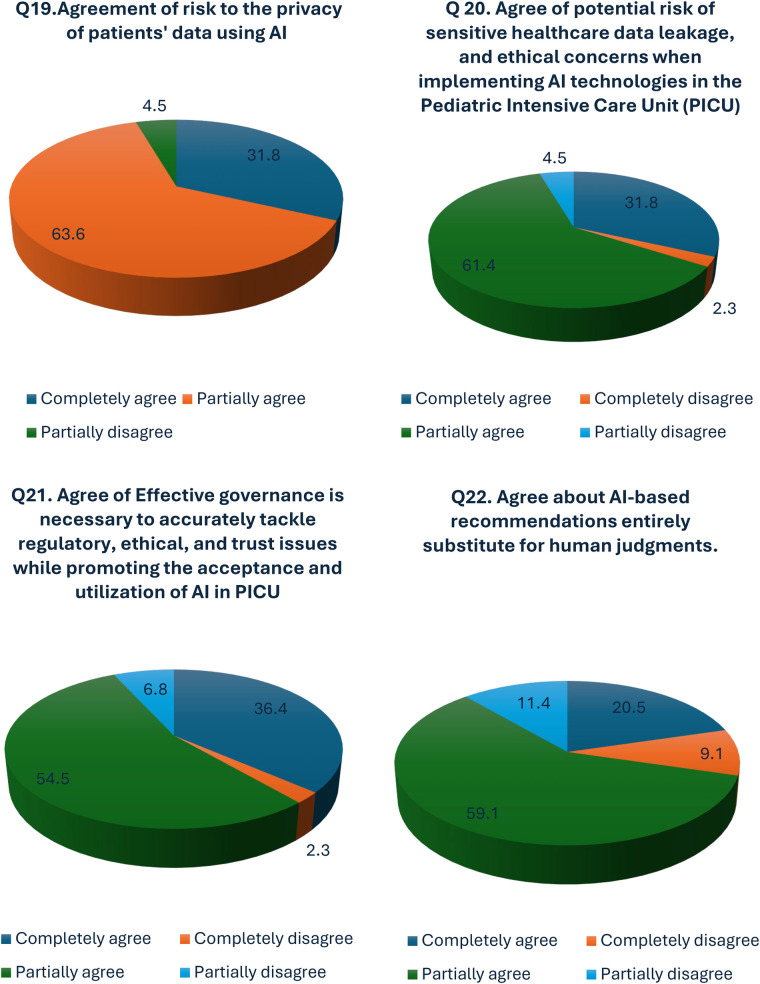
Perceptions of privacy risks, data leakage, ethical concerns, and trust issues related to AI in PICU.

Ethical concerns and patient information privacy are of utmost importance for health service providers. Therefore, we investigated the participants' opinions regarding the ethics and privacy of the patient data. The majority of the participants (63.6%) partially agreed to the fact that AI poses a risk to the privacy of patient data as opposed to 31.8% completely agreeing and a small number of participants (4.5%) completely disagreeing (*p*-value = 0.00) (Figure 3; Supplementary Table 1). Similarly, 93.2% (61.4% partially agreed and 3.18% completely agreed) of the participants expressed their concern over the potential risk of sensitive healthcare data leakage, and ethical concerns when implementing AI technologies in the PICU. The remaining 6.8% of the participants did not express concerns about privacy breaches while implementing AI in the PICU. Furthermore, 90.1% of the participants (54.5% partially in agreement and 36.4% fully in agreement) acknowledged that robust governance is essential for effectively addressing regulatory, ethical, and trust-related challenges while fostering the acceptance and implementation of AI in the PICU (*p*-value = 0.00) (Figure 3).

## Discussion

AI is increasingly recognized as a transformative force in healthcare (25). It can potentially enhance diagnostic accuracy, improve patient outcomes, and streamline clinical workflows (26, 27). By utilizing machine learning algorithms and data analytics, AI can help healthcare professionals make more informed decisions, identify patterns in complex data sets, and predict patient needs (4). This technology shows promise in routine tasks and advanced medical procedures, potentially revolutionizing various aspects of patient care and medical research. From personalized treatment plans to early disease detection, AI can significantly improve the standards of healthcare delivery, making it more efficient, precise, and accessible (28). As the applications of AI advance, its integration into healthcare systems is poised to address many existing challenges, paving the way for a future where AI-driven tools are an integral part of clinical practice (3).

Several studies were performed to harness the potential of Machine learning, and AI in PICU. The study conducted by Wang et al. found that diagnostic biomarkers, namely angiopoietin-1, angiopoietin-2, and bicarbonate, can effectively predict mortality in pediatric patients suffering from severe sepsis when admitted to PICU (29). By employing Support Vector Machine (SVM) technology, the research highlighted an indirect impact on clinical decision support systems. Similarly, Kennedy et al. found that developing and testing cardiac arrest prediction models improved the prediction accuracy for pediatric patients at risk of disability and death in the PICU. The use of SVM technology enabled the prediction of morbidity and mortality, enhancing early diagnostic capabilities and triage processes (30). A study found that a Crisis Standards of Care triage allocation framework could assess the necessity for mechanical ventilation, predict PICU length of stay, and evaluate mortality risks in pediatric patients. Utilizing linear regression for their analysis, the study provided insights into optimizing resource allocation and improving patient outcomes, achieving a Technology Readiness Level (TRL) of 7, which indicates a robust application of artificial intelligence in this area (31). Another study by Carlin et al. found that predicting individual physiologically acceptable states during PICU discharge offers valuable information regarding the total length of stay for pediatric patients (20). The study utilized a recursive neural network, thereby indirectly influencing care delivery and chronic management, and achieved a TRL of 8, indicating advanced applicability in clinical settings. Williams et al. found that analyzing medical data could predict critical factors such as length of stay, utilization of mechanical ventilation and inotropes, and mortality risk in PICU patients (21). The application of k-means clustering allowed for indirect contributions to clinical decision support, with the study achieving a TRL of 7, reflecting its established developmental status. Kim et al. established a model to predict all-cause mortality in the PICU (32).

Therefore, the present study aimed to understand the perception of healthcare providers regarding the utility of AI and the implementation of AI in PICUs to improve the outcomes of the patients. Implementing AI within PICU has elicited a spectrum of responses from healthcare professionals, as revealed through a detailed analysis of survey data. Respondents' familiarity with AI and Data Science showed significant differences, with a *p*-value of 0.00, indicating a notable variation in the levels of familiarity among the respondents, highlighting a significant variation in familiarity levels among participants (Figure 1). This discrepancy points to a potential gap in knowledge that may influence the perception and acceptance of AI within clinical settings. It is presumed that being a newer technology in the healthcare industry AI has yet to gain popularity despite its importance. The results of our study align with previous research (33–38). These studies collectively underscore the increasing acknowledgment of the potential of AI to enhance healthcare delivery and the accompanying challenges related to its implementation. These challenges encompass data privacy, ethical considerations, and the necessity for robust regulatory frameworks.

Despite initial apprehensions, most participants preferred the comprehensive or partial application of AI in managing patients within the PICU environment. This strong agreement suggests an optimistic view of the potential of AI to contribute positively to the medical field. Similarly, awareness about the potential AI applications in PICU varied significantly (*p*-value = 0.001), indicating a diverse level of awareness and perhaps an opportunity for educational initiatives to increase understanding of AI capabilities. This strong consensus on the benefits of AI aligns well with the ever-increasing interest of healthcare practitioners in leveraging technology to improve healthcare outcomes. Similar to our findings, a systematic review and meta-analyses showed approximately 22% of the studies indicated direct health improvements with AI, while others showcased AI's indirect outperformance of the traditional methods (39). Another study highlighted emerging themes such as health services management, predictive medicine, and clinical decision-making, with significant contributions from the US, China, and the UK, and the result indicated the role of AI in diagnosis, disease prediction, and personalized treatment paths, underscoring areas ripe for further exploration in data-intensive analysis and knowledge management for AI projects in healthcare (40).

The study confirmed high trust in AI-based decision support tools but revealed varied opinions on the effectiveness of AI in analyzing pediatric-specific data within the PICU. Our study found mixed opinions about AI's effectiveness in analyzing pediatric data in the PICU. Exploring the reasons behind this perception could be insightful, as it may be influenced by factors like complexity, dynamic patient interactions, and the significant impact of experienced clinicians on outcomes in clinical settings. Our study found widespread clinician support for early AI implementation, alongside significant concerns over patient data privacy and ethical issues (*p* < 0.001). There is strong consensus on the necessity of robust governance to address regulatory and trust challenges in pediatric intensive care settings, with high expectations for AI's rapid integration (*p* < 0.001).

On the other hand, a whopping 65% of the participants in our study stated that certain tasks, such as routine data management and record maintenance, could be delegated to AI (Figure 2). Therefore, it is highly likely that most clinicians and other healthcare providers would delegate AI to carry out routine patient monitoring and explore AI's agility and accuracy to improve patient care in PICU. One of the advantages of AI is the increased storage of health information and the facilitation of health data access, which is compatible with the use of AI to assist the daily management of professional activity and with the previously documented predisposition for delegating on AI the systematization of the patient's general symptomatology (41). Preferences for AI application in routine vs. advanced diagnostic tasks revealed notable significance (*p*-value = 0.050), suggesting a nuanced view of how best to deploy AI technologies within clinical workflows. The preference for AI-assisted monitoring systems over traditional methods was strongly supported (*p*-value = 0.000), alongside a recognition of AI's importance in improving PICU outcomes (*p*-value = 0.000). These viewpoints underscore the anticipated positive impact of AI on patient monitoring and overall care quality. A study suggests that AI can be integrated into various areas such as patient administration, clinical decision support, monitoring, and interventions to facilitate its meaningful implementation within a health system (42).

In our study, we observed that nurses reported greater familiarity with AI compared to other clinical professions. This could be attributed to their frequent interaction with AI-driven monitoring systems, routine involvement in patient care workflows, and exposure to training in advanced healthcare technologies. In addition, nurses in PICUs frequently engage directly with patient monitoring systems, many of which feature AI-driven components such as predictive analytics, alarms, and automated documentation tools. This hands-on experience may enhance their awareness of AI applications in clinical environments. Further, training and awareness programs were regularly organized for each module, covering instrument operations, recent advancements in healthcare technology, automated workflows, and data management. Nurses participated in these programs as part of their schedules, enhancing their knowledge of the latest developments in the healthcare sector.

These findings highlight the diverse viewpoints of healthcare professionals regarding the incorporation of AI in PICUs, emphasizing both its potential advantages and the challenges associated with its implementation. Despite initial concerns, there was a strong consensus on the positive impact of AI in enhancing patient care, particularly in routine tasks and advanced diagnostics. However, significant concerns about data privacy and ethical considerations were evident, underscoring the necessity for strong regulatory frameworks. Studies suggest clinicians are prepared to embrace AI, provided these challenges are effectively addressed. With the ongoing advancement of AI, its thoughtful integration into healthcare systems holds the promise of transforming pediatric critical care, improving patient outcomes, and bolstering clinical efficiency.

## Conclusion

The present study revealed that the healthcare practitioners engaged in the PICU favored using AI for routine monitoring of the patients. However, many also expressed reservations about employing AI for diagnoses and advanced healthcare. The healthcare sector acknowledges the potential of AI to enhance patient outcomes significantly. Nonetheless, concerns persist regarding data privacy, security breaches, and patient confidentiality in the context of AI implementation. It is crucial for all stakeholders, including policymakers, governments, the healthcare industry, and patients, to address these issues to ensure the proper and successful integration of AI in the PICU.

## Data Availability

The original contributions presented in the study are included in the article/Supplementary Material, further inquiries can be directed to the corresponding author.

## References

[1] Suleimenov IE Vitulyova YS Bakirov AS Gabrielyan OA. Artificial intelligence: what is it? In: Proceedings of the 2020 6th International Conference on Computer and Technology Applications (ICCTA ’20). New York, NY: Association for Computing Machinery (2020). p. 22–5. 10.1145/3397125.3397141

[2] Gomes B Ashley EA. Artificial intelligence in molecular medicine. N Engl J Med. (2023) 388:2456–65. 10.1056/NEJMra2204787 37379136

[3] Alowais SA Alghamdi SS Alsuhebany N Alqahtani T Alshaya AI Almohareb SN Revolutionizing healthcare: the role of artificial intelligence in clinical practice. BMC Med Educ. (2023) 23:689. 10.1186/s12909-023-04698-z 37740191 PMC10517477

[4] Davenport T Kalakota R. The potential for artificial intelligence in healthcare. Future Healthc J. (2019) 6:94–8. 10.7861/futurehosp.6-2-94 31363513 PMC6616181

[5] Rajpurkar P Chen E Banerjee O Topol EJ. AI in health and medicine. Nat Med. (2022) 28:31–8. 10.1038/s41591-021-01614-0 35058619

[6] Saqib M Iftikhar M Neha F Karishma F Mumtaz H. Artificial intelligence in critical illness and its impact on patient care: a comprehensive review. Front Med (Lausanne). (2023) 10:1176192. 10.3389/fmed.2023.1176192 37153088 PMC10158493

[7] Miller A. The future of health care could be elementary with Watson. CMAJ. (2013) 185:E367–8. 10.1503/cmaj.109-4442 23589429 PMC3680569

[8] Yoon JH Pinsky MR. Predicting adverse hemodynamic events in critically ill patients. Curr Opin Crit Care. (2018) 24:196–203. 10.1097/MCC.0000000000000496 29601321 PMC6007856

[9] Zimmerman JE Kramer AA Knaus WA. Changes in hospital mortality for United States intensive care unit admissions from 1988 to 2012. Crit Care. (2013) 17:R81. 10.1186/cc12695 23622086 PMC4057290

[10] Chen M Decary M. Artificial intelligence in healthcare: an essential guide for health leaders. Healthc Manag Forum. (2020) 33:10–8. 10.1177/0840470419873123 31550922

[11] Matheny ME Whicher D Thadaney Israni S. Artificial intelligence in health care: a report from the national academy of medicine. JAMA. (2020) 323:509–10. 10.1001/jama.2019.21579 31845963

[12] Paycha F. [Diagnosis with the aid of artificial intelligence: demonstration of the 1st diagnostic machine]. Presse Therm Clim. (1968) 105:22–5. 4910364

[13] Shah N Arshad A Mazer MB Carroll CL Shein SL Remy KE. The use of machine learning and artificial intelligence within pediatric critical care. Pediatr Res. (2023) 93:405–12. 10.1038/s41390-022-02380-6 36376506 PMC9660024

[14] Lonsdale H Jalali A Ahumada L Matava C. Machine learning and artificial intelligence in pediatric research: current state, future prospects, and examples in perioperative and critical care. J Pediatr. (2020) 221s:S3–10. 10.1016/j.jpeds.2020.02.039 32482232

[15] Masino AJ Harris MC Forsyth D Ostapenko S Srinivasan L Bonafide CP Machine learning models for early sepsis recognition in the neonatal intensive care unit using readily available electronic health record data. PLoS One. (2019) 14:e0212665. 10.1371/journal.pone.0212665 30794638 PMC6386402

[16] Medina R Bouhaben J de Ramón I Cuesta P Antón-Toro L Pacios J Electrophysiological brain changes associated with cognitive improvement in a pediatric attention deficit hyperactivity disorder digital artificial intelligence-driven intervention: randomized controlled trial. J Med Internet Res. (2021) 23:e25466. 10.2196/25466 34842533 PMC8665400

[17] Nimri R Battelino T Laffel LM Slover RH Schatz D Weinzimer SA Insulin dose optimization using an automated artificial intelligence-based decision support system in youths with type 1 diabetes. Nat Med. (2020) 26:1380–4. 10.1038/s41591-020-1045-7 32908282

[18] Clark MM Hildreth A Batalov S Ding Y Chowdhury S Watkins K Diagnosis of genetic diseases in seriously ill children by rapid whole-genome sequencing and automated phenotyping and interpretation. Sci Transl Med. (2019) 11:eaat6177. 10.1126/scitranslmed.aat6177 31019026 PMC9512059

[19] Shirwaikar RD. Estimation of caffeine regimens: a machine learning approach for enhanced clinical decision making at a neonatal intensive care unit (NICU). Crit Rev Biomed Eng. (2018) 46:93–115. 10.1615/CritRevBiomedEng.2018025933 30055527

[20] Carlin CS Ho LV Ledbetter DR Aczon MD Wetzel RC. Predicting individual physiologically acceptable states at discharge from a pediatric intensive care unit. J Am Med Inform Assoc. (2018) 25(12):1600–7. 10.1093/jamia/ocy122 30295770 PMC7647156

[21] Williams JB Ghosh D Wetzel RC. Applying machine learning to pediatric critical care data. Pediatr Crit Care Med. (2018) 19:599–608. 10.1097/pcc.0000000000001567 29727354

[22] Chaichulee S Villarroel M Jorge J Arteta C McCormick K Zisserman A Cardio-respiratory signal extraction from video camera data for continuous non-contact vital sign monitoring using deep learning. Physiol Meas. (2019) 40:115001. 10.1088/1361-6579/ab525c 31661680 PMC7655150

[23] Nagori A Dhingra LS Bhatnagar A Lodha R Sethi T. Predicting hemodynamic shock from thermal images using machine learning. Sci Rep. (2019) 9:91. 10.1038/s41598-018-36586-8 30643187 PMC6331545

[24] Väänänen A Haataja K Vehviläinen-Julkunen K Toivanen P. AI in healthcare: a narrative review. F1000Res. (2021) 10:6. 10.12688/f1000research.26997.2 PMC8577055 34804493

[25] Maleki Varnosfaderani S Forouzanfar M. The role of AI in hospitals and clinics: transforming healthcare in the 21st century. Bioengineering (Basel). (2024) 11:337. 10.3390/bioengineering11040337 38671759 PMC11047988

[26] Wang F Preininger A. AI In health: state of the art, challenges, and future directions. Yearb Med Inform. (2019) 28:16–26. 10.1055/s-0039-1677908 31419814 PMC6697503

[27] Olawade D Wada O Asaolu A Asaolu AJ Adereni T Ling J. Artificial intelligence in healthcare delivery: prospects and pitfalls. J Med Surg Public Health. (2024) 3:100108. 10.1016/j.glmedi.2024.100108

[28] Bohr A Memarzadeh K. The rise of artificial intelligence in healthcare applications. In: Bohr A Memarzadeh K, editors. *Artificial Intelligence in Healthcare*. Academic Press. (2020). p. 25–60. 10.1016/B978-0-12-818438-7.00002-2

[29] Wang K Bhandari V Giuliano JS Jr O Hern CS Shattuck MD Kirby M. Angiopoietin-1, angiopoietin-2 and bicarbonate as diagnostic biomarkers in children with severe sepsis. PLoS One. (2014) 9:e108461. 10.1371/journal.pone.0108461 25255212 PMC4178003

[30] Kennedy CE Turley JP. Time series analysis as input for clinical predictive modeling: modeling cardiac arrest in a pediatric ICU. Theor Biol Med Model. (2011) 8:40. 10.1186/1742-4682-8-40 22023778 PMC3213024

[31] Olive MK Owens GE. Current monitoring and innovative predictive modeling to improve care in the pediatric cardiac intensive care unit. Transl Pediatr. (2018) 7:120–8. 10.21037/tp.2018.04.03 29770293 PMC5938248

[32] Kim SY Kim S Cho J Kim YS Sol IS Sung Y A deep learning model for real-time mortality prediction in critically ill children. Crit Care. (2019) 23:279. 10.1186/s13054-019-2561-z 31412949 PMC6694497

[33] Castagno S Khalifa M. Perceptions of artificial intelligence among healthcare staff: a qualitative survey study. Front Artif Intell. (2020) 3:578983. 10.3389/frai.2020.578983 33733219 PMC7861214

[34] Orlova IA Akopyan ZA Plisyuk AG Tarasova EV Borisov EN Dolgushin GO Opinion research among Russian physicians on the application of technologies using artificial intelligence in the field of medicine and health care. BMC Health Serv Res. (2023) 23:749. 10.1186/s12913-023-09493-6 37442981 PMC10339534

[35] Abdullah R Fakieh B. Health care employees’ perceptions of the use of artificial intelligence applications: survey study. J Med Internet Res. (2020) 22:e17620. 10.2196/17620 32406857 PMC7256754

[36] Dorsey ER Topol EJ. Telemedicine 2020 and the next decade. Lancet. (2020) 395:859. 10.1016/S0140-6736(20)30424-4 32171399

[37] Özkaya G Aydin MO Alper Z. Distance education perception scale for medical students: a validity and reliability study. BMC Med Educ. (2021) 21:400. 10.1186/s12909-021-02839-w 34311725 PMC8311629

[38] Zeng X Zhang Y Kwong JS Zhang C Li S Sun F The methodological quality assessment tools for preclinical and clinical studies, systematic review and meta-analysis, and clinical practice guideline: a systematic review. J Evid Based Med. (2015) 8:2–10. 10.1111/jebm.12141 25594108

[39] Adegboro CO Choudhury A Asan O Kelly MM. Artificial intelligence to improve health outcomes in the NICU and PICU: a systematic review. Hosp Pediatr. (2022) 12:93–110. 10.1542/hpeds.2021-006094 34890453

[40] Secinaro S Calandra D Secinaro A Muthurangu V Biancone P. The role of artificial intelligence in healthcare: a structured literature review. BMC Med Inform Decis Mak. (2021) 21:125. 10.1186/s12911-021-01488-9 33836752 PMC8035061

[41] Fan W Liu J Zhu S Pardalos PM. Investigating the impacting factors for the healthcare professionals to adopt artificial intelligence-based medical diagnosis support system (AIMDSS). Ann Oper Res. (2020) 294:567–92. 10.1007/s10479-018-2818-y

[42] Reddy S Fox J Purohit MP. Artificial intelligence-enabled healthcare delivery. J R Soc Med. (2019) 112:22–8. 10.1177/0141076818815510 30507284 PMC6348559

[43] Whicher D Wu AW. Ethics review of survey research: a mandatory requirement for publication? Patient. (2015) 8:477–82. 10.1007/s40271-015-0141-0 26392006

[44] Oh S Kim JH Choi S-W Lee HJ Hong J Kwon SH. Physician confidence in artificial intelligence: an online mobile survey. J Med Internet Res. (2019) 21:e12422. 10.2196/12422 30907742 PMC6452288

