# Supplementary material for: Role of artificial intelligence in pediatric intensive care: a survey of healthcare staff perspectives in Saudi Arabia
Source: Front Pediatr. 2025 Feb 24;13:1533877. doi: 10.3389/fped.2025.1533877 (PMC11891184; doi:10.3389/fped.2025.1533877)
Supplement: Supplementary file 1 [file Datasheet1.docx]

**Supplementary Table 1.** The questionnaire used in the study.

Understanding Opinions and Practices: Surveying Artificial Intelligence Adoption in Pediatric ICU Care

1. **Age (in Years)**

*Mark only one oval.*


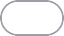
 20-29


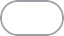
 30-39


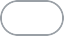
 40-49


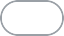
 50-59


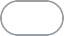
 60-69


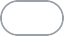
 70 and above

1. **Gender**

*Mark only one oval.*


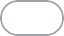
 Male
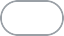
 Female

1. **What is your profession?**

*Mark only one oval.*

Doctor Nurse Clinician Technician Therapist Dieticians Manager Other:

1. **Professional experience (in Years)**

*Mark only one oval.*


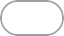
 <1


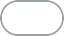
 1-9


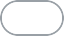
 10-19


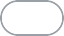
 20 and above

1. **Professional practice**

*Mark only one oval.*

Govt. Hospitals Private Hospitals

Independent practitioner

Other:

1. **How familiar are you with the use of Internet in your profession?**

*Mark only one oval.*


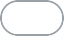
 Very familiar


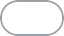
 Somewhat familiar
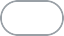
 Not familiar at all

1. **Are you familiar with Artificial Intelligence (AI), Data Science?**

*Mark only one oval.*


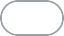
 Very familiar


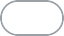
 Somewhat familiar
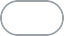
 Not familiar at all

1. **Do you agree AI will improve medical specialty?**

*Mark only one oval.*


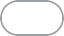
 Completely agree
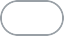
 Partially agree
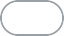
 Partially disagree


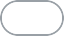
 Completely disagree

1. **How familiar are you with the potential applications of AI in pediatric ICU settings?**

*Mark only one oval.*


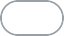
 Very familiar


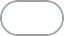
 Somewhat familiar
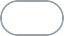
 Not familiar at all

1. **How useful do you think AI could be in your area of Pediatric ICU?**

*Mark only one oval.*


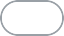
 Extremely useful
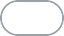
 Somewhat useful
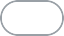
 not useful

1. **Are you aware of any specific AI applications currently used or tested in pediatric ICU settings?**

*Mark only one oval.*


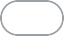
 Yes
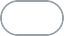
 No

1. **Would you trust AI-based decision support tools in pediatric ICU management?**

*Mark only one oval.*


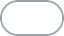
 Yes, completely
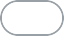
 Yes, partially
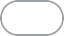
 No, not at all

1. **Do you think AI can effectively analyze pediatric-specific data and trends in the ICU?**

*Mark only one oval.*


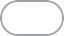
 Yes
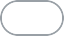
 No


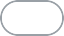
 Not sure

1. **Do you agree AI will simplify the patient care management in pediatric ICU setting?**

*Mark only one oval.*


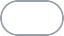
 Completely agree
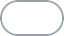
 Partially agree
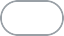
 Partially disagree


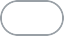
 Completely disagree

1. **Do you agree AI will increase the accuracy of the diagnostics results in pediatric ICU setting?**

*Mark only one oval.*


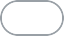
 Completely agree
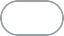
 Partially agree
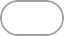
 Partially disagree


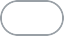
 Completely disagree

1. **Would you prefer implementing AI in performing the routine tasks in pediatric ICU setting or advanced level diagnostic analysis?**

*Mark only one oval.*


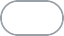
 Routine task, data management, records maintenance
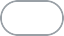
 Advance level diagnostic purpose

1. **Would you prefer AI-assisted monitoring systems for pediatric ICU patients over traditional monitoring methods?**

*Mark only one oval.*


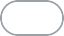
 Yes
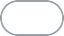
 No

1. **How important do you think AI is for improving outcomes in pediatric ICU patients?**

*Mark only one oval.*


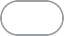
 Extremely important
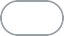
 Moderately important
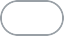
 Not important

1. **How much do you agree with the following statement: There is a risk of privacy issue of patients data using AI.**

*Mark only one oval.*


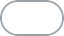
 Completely agree
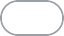
 Partially agree
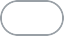
 Partially disagree


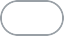
 Completely disagree

1. **How much do you agree with the following statement?**

There is potential risk of sensitive healthcare data leakage, ethical concerns, when implementing AI technologies in the Pediatric Intensive Care Unit (PICU).

*Mark only one oval.*


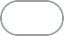
 Completely agree
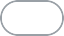
 Partially agree
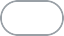
 Partially disagree


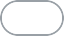
 Completely disagree

1. **How much do you agree with the following statement?**

Effective governance is necessary to accurately tackle regulatory, ethical, and trust issues while promoting the acceptance and utilization of AI in PICU.

*Mark only one oval.*


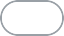
 Completely agree
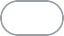
 Partially agree
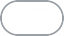
 Partially disagree


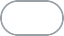
 Completely disagree

1. **How much do you agree with the following statement?**

AI-based recommendations entirely substitute for human judgements.

*Mark only one oval.*


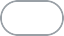
 Completely agree
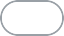
 Partially agree
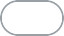
 Partially disagree


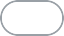
 Completely disagree

1. **In your opinion, how quickly do you expect AI to become a standard part of pediatric ICU care?**

*Mark only one oval.*


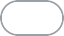
 Within the next few years
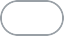
 Within the next decade


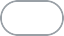
 More than a decade from now

**Supplementary Table 2.** Statistical significance results of implementing AI in PICU settings based on a non-parametric (chi-square) test.

| **Question No.** | **Questions** | **Answer** | ***p*-value** |
| --- | --- | --- | --- |
| 7 | Are you familiar with Artificial Intelligence (AI), Data Science? | - Very familiar - Somewhat familiar - Not familiar at all | 0.000 |
| 8 | Do you agree AI will improve the medical specialty? | - Completely agree - Partially agree - Partially disagree - Completely disagree | 0.004 |
| 9 | How familiar are you with the potential applications of AI in paediatric ICU settings? | - Very familiar - Somewhat familiar - Not familiar at all | 0.001 |
| 10 | How useful do you think AI could be in your area of Paediatric ICU? | - Extremely useful - Somewhat useful - not useful | 0.000 |
| 11 | Are you aware of any specific AI applications currently used or tested in paediatric ICU settings? | - Yes - No | 0.050 |
| 12 | Would you trust AI-based decision support tools in paediatric ICU management? occur with equal probabilities. | - Yes, completely - Yes, partially - No, not at all | 0.000 |
| 13 | Do you think AI can effectively analyse paediatric-specific data and trends in the ICU? | - Yes - No - Not sure | 0.291 |
| 14 | Do you agree AI will simplify patient care management in Paediatric ICU? | - Completely agree - Partially agree - Partially disagree - Completely disagree | 0.000 |
| 15 | Do you agree AI will increase the accuracy of the diagnostics results in paediatric ICU settings? | - Completely agree - Partially agree - Partially disagree - Completely disagree | 0.000 |
| 18 | Would you prefer implementing AI in routine tasks in a paediatric ICU setting or advanced-level diagnostic analysis? | - Routine task, data management, records maintenance - Advance level diagnostic purpose | 0.050 |
| 17 | Would you prefer AI-assisted monitoring systems for pediatric ICU patients over traditional monitoring methods? | - Yes - No | 0.000 |
| 18 | How important do you think AI is for improving outcomes in paediatric ICU patients? | - Extremely important - Moderately important - Not important | 0.000 |
| 19 | How much do you agree with the following statement: There is a risk to the privacy of patients' data using AI? | - Completely agree - Partially agree - Partially disagree - Completely disagree | 0.000 |
| 20 | How much do you agree with the following statement?  There is a potential risk of sensitive healthcare data leakage, and ethical concerns when implementing AI technologies in the Paediatric Intensive Care Unit (PICU). | - Completely agree - Partially agree - Partially disagree - Completely disagree | 0.000 |
| 21 | How much do you agree with the following statement?  Effective governance is necessary to accurately tackle regulatory, ethical, and trust issues while promoting the acceptance and utilization of AI in PICU. | - Completely agree - Partially agree - Partially disagree - Completely disagree | 0.000 |
| 22 | How much do you agree with the following statement?  AI-based recommendations entirely substitute for human judgments. occur with equal probabilities. | - Completely agree - Partially agree - Partially disagree - Completely disagree | 0.000 |
| 23 | In your opinion, how quickly do you expect AI to become a standard part of paediatric ICU care? occur with equal probabilities. | - Within the next few years - Within the next decade - More than a decade from now | 0.000 |

p-values are based on a non-parametric (chi-square) test.
